# A Bayesian Perspective on the Reproducibility Project: Psychology

**DOI:** 10.1371/journal.pone.0149794

**Published:** 2016-02-26

**Authors:** Alexander Etz, Joachim Vandekerckhove

**Affiliations:** 1 Department of Psychology, University of Amsterdam, Amsterdam, the Netherlands; 2 Department of Cognitive Sciences, University of California, Irvine, Irvine, CA, United States of America; 3 Department of Statistics, University of California, Irvine, Irvine, CA, United States of America; Universiteit Gent, BELGIUM

## Abstract

We revisit the results of the recent Reproducibility Project: Psychology by the Open Science Collaboration. We compute Bayes factors—a quantity that can be used to express comparative evidence for an hypothesis but also for the null hypothesis—for a large subset (*N* = 72) of the original papers and their corresponding replication attempts. In our computation, we take into account the likely scenario that publication bias had distorted the originally published results. Overall, 75% of studies gave qualitatively similar results in terms of the amount of evidence provided. However, the evidence was often weak (i.e., Bayes factor < 10). The majority of the studies (64%) did not provide strong evidence for either the null or the alternative hypothesis in either the original or the replication, and no replication attempts provided strong evidence in favor of the null. In all cases where the original paper provided strong evidence but the replication did not (15%), the sample size in the replication was smaller than the original. Where the replication provided strong evidence but the original did not (10%), the replication sample size was larger. We conclude that the apparent failure of the Reproducibility Project to replicate many target effects can be adequately explained by overestimation of effect sizes (or overestimation of evidence against the null hypothesis) due to small sample sizes and publication bias in the psychological literature. We further conclude that traditional sample sizes are insufficient and that a more widespread adoption of Bayesian methods is desirable.

## 1 Introduction

The summer of 2015 saw the first published results of the long-awaited Reproducibility Project: Psychology by the Open Science Collaboration [1] (henceforth OSC). In an attempt to closely replicate 100 studies published in leading journals, fewer than half were judged to successfully replicate. The replications were pre-registered in order to avoid selection and publication bias and were evaluated using multiple criteria. When a replication was judged to be successful if it reached statistical significance (i.e., *p* <.05), only 39% were judged to have been successfully reproduced. Nevertheless, the paper reports a.51 correlation between original and replication effect sizes, indicating some degree of robustness of results (see their Fig 3).

Much like the results of the project, the reactions in media and social media have been mixed. In a first wave of reactions, headlines ranged from the dryly descriptive “Scientists replicated 100 psychology studies, and fewer than half got the same results” [2] and “More than half of psychology papers are not reproducible” [3] to the crass “Study reveals that a lot of psychology research really is just ‘psycho-babble’” [4]. A second wave of reactions shortly followed. Editorials with titles such as “Psychology is not in crisis” [5] and a statement by the American Psychological Association [6] were quick to emphasize the possibility of many hidden moderators that rendered the replications ineffective. OSC acknowledges this: “unanticipated factors in the sample, setting, or procedure could still have altered the observed effect magnitudes,” but it is unclear what, if any, bearing this has on the robustness of the theories that the original publications supported.

In addition to the unresolved possibility of hidden moderators, there is the issue of lacking statistical power. The statistical power of an experiment is the frequency with which it will yield a statistically significant effect in repeated sampling, assuming that the underlying effect is of a given size. All other things—such as the design of the study and the true size of the effect—being equal, statistical power is determined by an experiment’s sample size. Low-powered research designs undermine the credibility of statistically significant results in addition to increasing the probability of nonsignificant ones (see [7] and the references therein for a detailed argument); furthermore, low-powered studies generally provide only small amounts of evidence (in the form of weak Bayes factors; see below).

Among the insights reported in OSC is that “low-power research designs combined with publication bias favoring positive results together produce a literature with upwardly biased effect sizes,” and that this may explain why replications—unaffected by publication bias—show smaller effect sizes. Here, we formally evaluate that insight, and use the results of the Reproducibility Project: Psychology to conclude that publication bias and low-powered designs indeed contribute to the poor reproducibility, but also that many of the replication attempts in OSC were themselves underpowered. While the OSC aimed for a minimum of 80% power (with an average of 92%) in all replications, this estimate was based on the observed effect size in the original studies. In the likely event that these observed effect sizes were inflated (see next section), the sample size recommendations from prospective power analysis will have been underestimates, and thus replication studies will tend to find mostly weak evidence as well.

### 1.1 Publication bias

Reviewers and editors in psychology journals are known to put a premium on ‘positive’ results. That is, they prefer studies in which a statistically significant result is used to support the existence of an effect. Nearly six decades ago, Sterling [8] noted this anomaly in the public record: In four prominent psychology journals, 95 to 99% of studies that performed a significance test rejected the null hypothesis (i.e., $H_{0}$). Sterling concludes by noting two key findings, “Experimental results will be printed with a greater probability if the relevant test of significance rejects $H_{0}$,” and, “The probability that an experimental design will be replicated becomes very small once such an experiment appears in print” (p. 33).

Moreover, it is a truism that studies published in the psychology literature are only a subset of the studies psychologists conduct, and various criteria are used to determine if a study should be published in a given journal. Studies that do not meet the criteria are relegated to lab file drawers [9]. A selective preference for publishing studies that reject $H_{0}$ is now known as *publication bias*, and is recognized as one cause of the current crisis of confidence in psychology [10].

When journals selectively publish only those studies that achieve statistical significance, average published effect sizes inevitably inflate because the significance threshold acts as a filter; only the studies with the largest effect sizes have sufficiently low *p*-values to make it through to publication. Studies with smaller, non-significant effects are rarely published, driving up the average effect size [11]. Readers who wish to evaluate original findings and replications alike must take into account the fact that our “very publication practices themselves are part and parcel of the probabilistic processes on which we base our conclusions concerning the nature of psychological phenomena” [12] (p. 427). Differently put, the publication criteria should be considered part of the experimental design [13]. For the current project, we choose to account for publication bias by modeling the publication process as a part of the data collection procedure, using a Bayesian model averaging method proposed by Guan and Vandekerckhove [14] and detailed in Section 2.2 below.

## 2 Methods

### 2.1 The Bayes factor

To evaluate replication success we will make use of *Bayes factors* [15, 16]. The Bayes factor (*B*) is a tool from Bayesian statistics that expresses how much a data set shifts the balance of evidence from one hypothesis (e.g., the null hypothesis $H_{0}$) to another (e.g., the alternative hypothesis $H_{A}$). Bayes factors require researchers to explicitly define the models under comparison.

In this report we compare the null hypothesis of no difference against an alternative hypothesis with a potentially nonzero effect size. Our prior expectation regarding the effect size under $H_{A}$ is represented by a normal distribution centered on zero with variance equal to 1 (this is a *unit information prior*, which carries a weight equivalent to approximately one observation [17]).

Other analysts could reasonably choose different prior distributions when assessing these data, and it is possible they would come to different conclusions. For example, in the case of a replication study specifically, a reasonable choice for the prior distribution of $H_{A}$ is the posterior distribution of the originally reported effects [18]. Using the original study’s posterior as the replication’s prior asks the question, “Does the result from the replication study fit better with predictions made by a null effect or by the originally reported effect?” A prior such as this would lend itself to more extreme values of the Bayes factor because the two hypotheses make very different predictions; the null hypothesis predicts replication effect sizes close to zero, whereas the original studies’ posterior distributions will typically be centered on relatively large effect sizes and hence predict large replication effect sizes. As such, Bayes factors for replications that find small-to-medium effect sizes will often favor $H_{0}$ (*δ* = 0) over the alternative model that uses the sequential prior because the replication result poorly fits the predictions made by the original posterior distribution, whereas small-to-medium effects will yield less forceful evidence in favor of $H_{0}$ over the alternative model using the unit information prior that we apply in this analysis.

There are two main reasons why, in the present paper, we choose to use the unit information prior over this sequential prior. First, our goal is not to evaluate how well empirical results reproduce, but rather to see how the *amount of evidence* gathered in an original study compares to that found in an *independent* replication attempt. This question is uniquely addressed by computing Bayes factors on two data sets, using identical priors. Compared to the sequential prior, the unit information prior we have chosen for our analysis is somewhat conservative, meaning that it requires more evidence before strongly favoring $H_{0}$ in a replication study. Indeed, results presented in a blog post by the first author [19] suggest that when a sequential prior is used approximately 20% of replications show strong evidence favoring $H_{0}$, as opposed to no replications strongly favoring $H_{0}$ with the unit information prior used in this report. Of course, it is to be expected that different analysts obtain different answers with different priors, because they are asking different questions (as Sir Harold Jeffreys [20] famously quipped: “It is sometimes considered a paradox that the answer depends not only on the observations but on the question; it should be a platitude,” p. vi).

A second reason we do not use the sequential prior in this report is that it does not take into account publication bias. Assuming that publication bias has a greater effect on the original studies than it did on the (pre-registered, certain to be published regardless of outcome) replications, the observed effect sizes in original and replicate studies are not expected to be equal. Using the original posterior distribution as a prior in the replication study would penalize bias in the original result; since the replication attempts will nearly always show smaller effect sizes than the biased originals, it will be more common to ‘fail to replicate’ these original findings (by accumulating evidence in favor of $H_{0}$ in the replication). However, here we are interested in evaluating the evidential support for the effects in the replication, rather than using them to quantify the effect of publication bias. In other words, we are interested in answering the following question: If we treat the two results as independent, do they provide similar degrees of evidence?

#### Interpretation of the Bayes factor

The Bayes factor is most conveniently interpreted as the degree to which the data sway our belief from one to the other hypothesis. In a typical situation, assuming that the reader has no reason to prefer the null hypothesis over the alternative before the study (i.e., 1:1 odds, or both have a prior probability of .50), a Bayes factor of 3 in favor of the alternative will change their odds to 3:1 or a posterior probability of .75 for $H_{A}$. Since a Bayes factor of 3 would carry a reader from equipoise only to a 75% confidence level, we take this value to represent only weak evidence. Put another way, accepting a 75% posterior probability for $H_{A}$ means that the reader would accept a one-in-four chance of being wrong. To put that in a context: that is the probability of correctly guessing the suit of a randomly-drawn card; and the researcher would reasonably prefer to bet on being wrong than on a fair die coming up six. That is to say, it is evidence that would not even be convincing to an uninvested reader, let alone a skeptic (who might hold, say, 10:1 prior odds against $H_{A}$). Table 1 provides posterior probabilities associated with certain Bayes factors *B* assuming prior odds of 1:1. In that table, we have also added some descriptive labels for Bayes factors of these magnitudes (these labels are similar in spirit to those suggested by Jeffreys [20]). Finally, it bears pointing out that if a researcher wants to move the probability of $H_{0}$ from 50% to below 5%, a Bayes factor of at least 19 is needed.

**Table 1:** Descriptive labels for certain Bayes factors.

Label	*B*	$p\left(H_{A}|\text{data}\right)$^a^
Strongly support $H_{A}$	10	91%
Weakly support $H_{A}$	3	75%
Ambiguous information	1	50%
Weakly support $H_{0}$	13	25%
Strongly support $H_{0}$	110	9%

^a^: $p(H_{A}|\text{data})$ is the posterior probability of $H_{A}$ assuming prior equiprobability between $H_{0}$ and $H_{A}$.

It is important to keep in mind that the Bayes factor as a measure of evidence must always be interpreted in the light of the substantive issue at hand: For extraordinary claims, we may reasonably require more evidence, while for certain situations—when data collection is very hard or the stakes are low—we may satisfy ourselves with smaller amounts of evidence. For our purposes, we will only consider Bayes factors of 10 or more as evidential—a value that would take an uninvested reader from equipoise to a 91% confidence level (a level at which an unbiased, rational reader is willing to bet up to ten cents on $H_{A}$ to win back one cent if they are right). Since the Bayes factor represents the evidence from the sample, readers can take these Bayes factors and combine them with their own personal prior odds to come to their own conclusions.

### 2.2 Mitigation of publication bias

The academic literature is unfortunately biased. Since studies in which the null hypothesis is confidently rejected are published at a higher rate than those in which it is not, the literature is “unrepresentative of scientists’ repeated samplings of the real world” [21]. A retrospective analysis of published studies must therefore take into account the fact that these studies are somewhat exceptional in having passed the so-called *statistical significance filter* [11].

**Fig 1:**
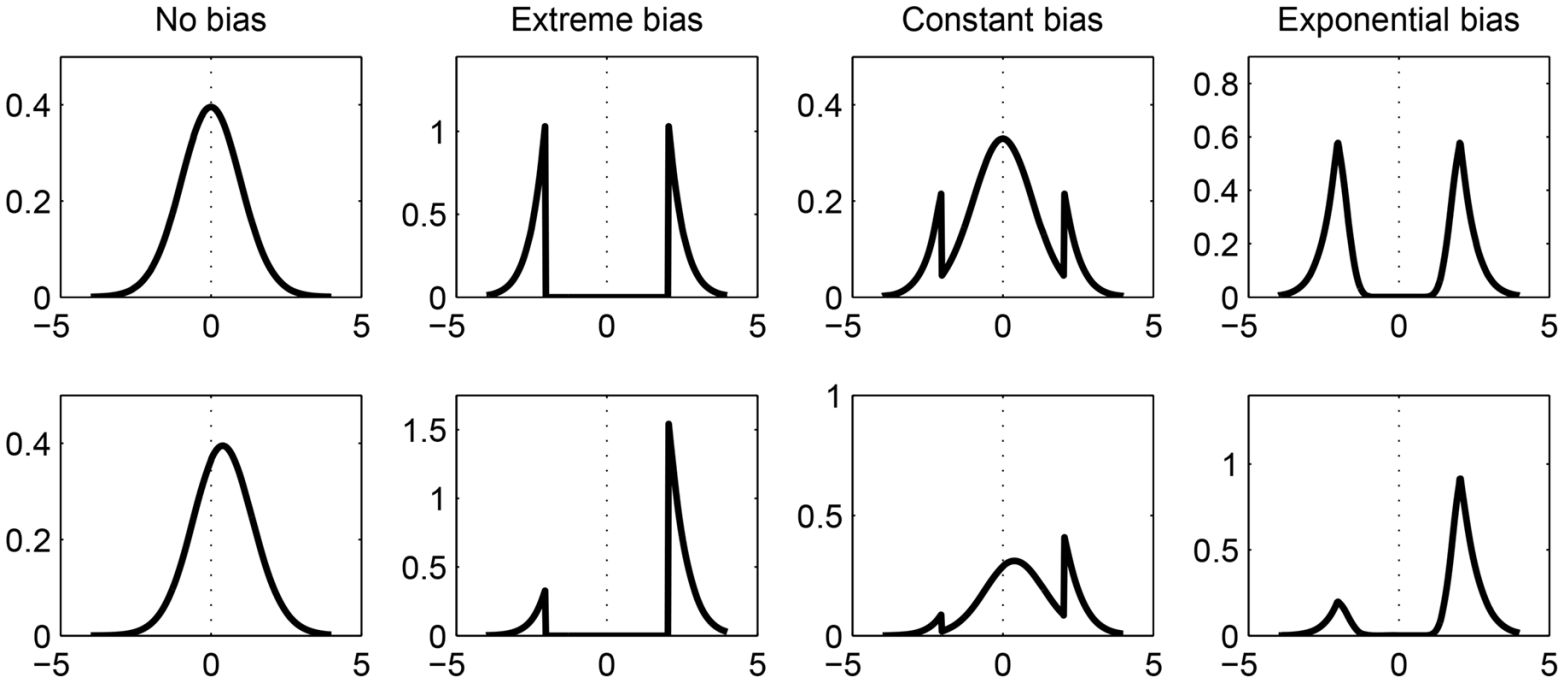
Predicted distributions of *t* statistics in the literature. Predicted distributions are shown under the four censoring mechanisms we consider (columns) and two possible states of nature (top row: $H_{0}$ true (*δ* = 0); bottom row: $H_{0}$ false (*δ* ≠ 0)).

Guan and Vandekerckhove [14] define four censoring functions that serve as models of the publication process. Each of these censoring functions formalizes a statistical significance filter, and each implies a particular *expected distribution of test statistics that make it to the literature*. The first, a *no-bias model*, where significant and non-significant results are published with equal probability, implies the typical central and non-central *t* distributions (for null and non-null effects, respectively). The second, an *extreme-bias model*, indexes a process where non-significant results are never published. This model assigns nonzero density only to regions where significant results occur (i.e., *p* <.05) and nowhere else. The third, a *constant-bias model*, indexes a process where non-significant results are published at a rate that is some constant *π* (0 ≤ *π* ≤ 1) times the rate at which significant results are published. These distributions look like typical *t* distributions but with the central (non-significant) region weighted down, creating large spikes in density over critical regions in the *t*-distribution. The fourth, an *exponential-bias model*, indexes a process where the probability that non-significant results are published decreases exponentially as (*p* − *α*) increases (i.e., “marginally significant” results have a moderately high chance of being published). These distributions have spikes in density around critical *t*-values. Fig 1 shows the predicted distribution of published *t* values under each of the four possible censoring functions, with and without a true effect.

None of these censoring functions are likely to capture the exact nature of the publication process in all of the cases we consider, but we believe they span a reasonable range of possible processes. Assuming that these four models reasonably represent possible statistical significance filters, we can use a Bayesian model averaging method to compute a single *mitigated Bayes factor* (*B*^*M*^) that takes into account that a biased process may have led to the published effect. The procedure essentially serves to raise the evidentiary bar for published studies if publication bias was not somehow prevented (e.g., through pre-registration). A unique feature of this method (compared to other bias mitigation methods such as PET-PEESE [22]) is that it allows us to quantify mitigated evidence for or against the null hypothesis on a continuous scale—a feature that will become useful when we compare original and replicated studies, below.

#### Calculation of the mitigated Bayes factor

To calculate *B*^*M*^, we first define a likelihood function in which the *t* distribution is multiplied by a weighting function *w*, so that

$$\begin{array}{c} p_{w}^{+}\left(x|n,\delta ,\theta \right)\propto t_{n}\left(x|\delta \right)w\left(x|\theta \right). \end{array}$$ (1)

Here, *x* is the reported *t*-value, *n* stands for the associated degrees of freedom, *δ* is the effect size parameter of the noncentral *t* distribution, and *w* is one of the four censoring functions which has optional parameters *θ* (see Table 2 for details regarding weighting functions). Eq (1) describes four possible models, each with some effect size *δ*. Together, these four models form the alternative hypothesis $H_{A}$. We construct four additional models in which *δ* = 0 (i.e., there is no underlying effect): $p_{w}^{-}\left(x|n,\theta \right)=p_{w}^{+}\left(x|n,\delta =0,\theta \right)$. Here the *t* distribution reduces to the central *t*, and these four models together form the null hypothesis $H_{0}$.

**Table 2:** The four weighting functions.

Model	Weight *w* if *p* >.05	Parameters *θ*
No bias	*w*(*x*) = 1	None
Extreme bias	*w*(*x*) = 0	None
Constant bias	*w*(*x*|*π*) = *π*	*π*
Exponential bias	*w*(*x*|*λ*) = *e*^(−*λ*(*p*−.05))^	*λ*

Note: *w*(*x*) is always 1 for results that are statistically significant at the.05-level. The dependency on the design and data properties that determine statistical significance is implied.

Second, we obtain the Bayesian evidences $E_{w}^{+}$ and $E_{w}^{-}$ by integrating the likelihood for each model over the prior:

$$\begin{array}{ccc} E_{w}^{+} & = & \int_{\Theta }\int_{\Delta }p_{w}^{+}\left(x|n,\delta ,\theta \right)p\left(\delta \right)p\left(\theta \right)d\delta d\theta \\ E_{w}^{-} & = & \int_{\Theta }p_{w}^{-}\left(x|n,\theta \right)p\left(\theta \right)d\theta . \end{array}$$

$E_{w}^{+}$ and $E_{w}^{-}$ are also known as the *marginal likelihoods* of these models (i.e., the probability density of the data under the model, as a prior-weighted average over all possible parameter constellations), and they can be conveniently approximated with Gaussian quadrature methods [23].

**Fig 2:**
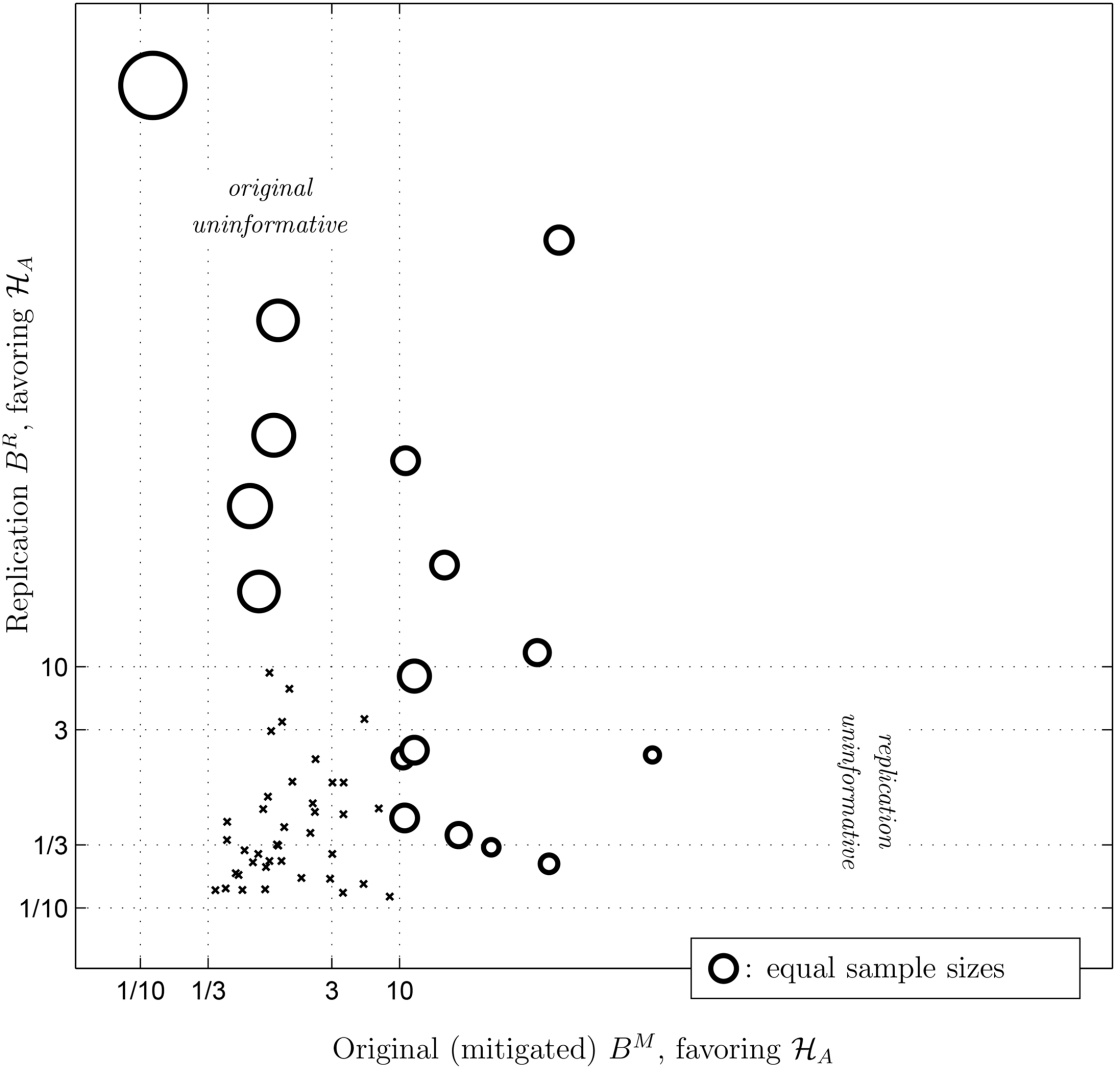
Evidence resulting from replicated studies plotted against evidence resulting from the original publications. For the original publications, evidence for the alternative hypothesis was calculated taking into account the possibility of publication bias. Small crosses indicate cases where neither the replication nor the original gave strong evidence. Circles indicate cases where one or the other gave strong evidence, with the size of each circle proportional to the ratio of the replication sample size to the original sample size (a reference circle appears in the lower right). The area labeled ‘replication uninformative’ contains cases where the original provided strong evidence but the replication did not, and the area labeled ‘original uninformative’ contains cases where the reverse was true. Two studies that fell beyond the limits of the figure in the top right area (i.e., that yielded extremely large Bayes factors both times) and two that fell above the top left area (i.e., large Bayes factors in the replication only) are not shown. The effect that relative sample size has on Bayes factor pairs is shown by the systematic size difference of circles going from the bottom right to the top left. All values in this figure can be found in S1 Table.

Finally, the posterior probability of each hypothesis can be calculated by (1) multiplying each evidence value with the corresponding model prior (where a ‘model’ is any one of the eight possible combinations of weighting function *w* and the null or alternative hypothesis; see Fig 1); (2) dividing each of those products with the sum of all such products for all models; and (3) summing the posterior probabilities for all models within an hypothesis. This can be rearranged to yield the following expression for the posterior:

$$\begin{array}{c} Pr\left(H_{A}|x\right)=Pr\left(H_{A}\right)\times \frac{\sum_{w}Pr\left(w\right)E_{w}^{+}}{\sum_{k}Pr\left(k\right)\left[Pr\left(H_{A}\right)E_{k}^{+}+Pr\left(H_{0}\right)E_{k}^{-}\right]}, \end{array}$$

where *Pr*(*w*) is the prior probability of censoring function *w* and $Pr(H_{A})$ is the prior probability that there is a nonzero effect. To obtain the Bayes factor, we restate in terms of posterior and prior ratios to obtain the simple expression:

$$\begin{array}{c} \underset{\text{Posterior odds}}{\underset{︸}{\frac{Pr(H_{A}|x)}{Pr(H_{0}|x)}}}=\underset{\text{Prior odds}}{\underset{︸}{\frac{Pr(H_{A})}{Pr(H_{0})}}}\;\times \underset{\text{Mitigated Bayes factor}}{\underset{︸}{\frac{\sum_{w}Pr\left(w\right)E_{w}^{+}}{\sum_{w}Pr\left(w\right)E_{w}^{-}}}}, \end{array}$$

where the second factor on the right hand side now represents the mitigated Bayes factor *B*^*M*^. Full details and MATLAB/Octave code to implement the procedure can be found here: http://bit.ly/1Nph9xQ.

### 2.3 Sample

We limited our analysis to studies that relied on univariate tests in order to apply the statistical mitigation method developed by Guan and Vandekerckhove [14]. A total of *N* = 72 studies were eligible. This includes all studies that relied on *t*-tests, univariate *F*-tests, and univariate regression analyses. This limits the generality of our conclusions to these cases, which fortunately constitute the bulk of studies in the Reproducibility Project: Psychology. A list of included studies and their inferential statistics is provided in S1 Table. Additionally, we conducted a sensitivity analysis varying the scale of the prior distribution among reasonable values (.5 to 2.0); this revealed no concerns that affect the conclusions or recommendations of the present analysis.

## 3 Results

### 3.1 Evidence in the original studies, taken at face value

For the original studies, we first computed “face value” Bayes factors that do not take into account the possibility of a biased publication process. By this measure, we find that 31 of the original studies (43%) provide strong support for the alternative hypothesis (*B* ≥ 10). No studies provide strong evidence for the null hypothesis. The remaining 57% provide only weak evidence one way or the other.

The small degrees of evidence provided by these published reports, taken at face value, are consistent with observations by Wetzels et al. [24] as well as the cautionary messages by Johnson [25] and Maxwell, Lau, and Howard [26].

### 3.2 Evidence in the original studies, corrected for publication bias

When we apply the statistical mitigation method of Guan and Vandekerckhove [14], the evidence for effects generally shrinks. After correction for publication bias, only 19 (26%) of the original publications afford strong support for the alternative hypothesis (*B*^*M*^ ≥ 10). A sizable majority of studies (53, or 74%) provide only ambiguous or weak information, with none finding strong evidence in favor of the null.

### 3.3 Evidence in the replication studies

The set of replication studies was entirely preregistered, with all data sets fully in the open and no opportunity for publication bias to muddy the results. Hence, no mitigation of bias is called for. Of the 72 replication studies, 15 (21%) strongly support the alternative hypothesis (*B*^*R*^ ≥ 10) and none strongly support the null. Twenty-seven (38%) provide only ambiguous information, and another 25 (35%) provide weak evidence for the null hypothesis.

### 3.4 Consistency of results

One of the stated goals of the Reproducibility Project: Psychology was to test whether previously found effects would obtain in an identical replication of a published study. Focusing on Bayesian evidence, we can now evaluate whether similar studies support similar conclusions. In 46 cases (64%), neither the original study nor the replication attempt yielded strong evidence (i.e., *B* ≥ 10). In only 8 cases (11%) did both the original study and the replication strongly support the alternative hypothesis. In 11 cases (15%) the original study strongly supported the alternative but the replication did not, and in 7 cases (10%) the replication provided strong evidence for the alternative whereas the original did not. The frequencies of these Bayes factor transitions are given in Table 3.

**Table 3:** Consistency of Bayes factors across original and replicate studies. Columns indicate the magnitude of the mitigated Bayes factor from the original study, and rows indicate the magnitude of the Bayes factor obtained in the replication project.

		*Mitigated Bayes factor (original study)*					
		0 − 1/10	1/10 − 1/3	1/3 − 3	3 − 10	10 − ∞	*sum*
*Replication study “face-value” Bayes factor*	0 − 1/10	0	0	0	0	0	0
	1/10 − 1/3	0	0	18	4	3	25
	1/3 − 3	0	0	16	4	7	27
	3 − 10	0	0	3	1	1	5
	10 − ∞	0	1	6	0	8	15
	*sum*	0	1	43	9	19	72

Fig 2 shows (in logarithmic coordinates) the Bayes factor of the replication *B*^*R*^ plotted against the bias-corrected Bayes factor of the original result *B*^*M*^. The majority of cases in which neither the original nor the replication provided strong evidence are displayed as the cluster of small crosses in the lower left of the figure. Circles represent cases where at least one of the attempts yielded strong evidence.

The observation that there are only 8 cases where both original and replication find strong evidence for an effect, while there are 18 cases in which one does and the other does not, seems at first to indicate a large degree of inconsistency between pairs of otherwise similar studies. To explain this inconsistency, Fig 2 highlights a major difference between each original and replication: The chosen sample size. The size of the circles indicates the ratio of the replication sample size to the original sample size. In each of the 11 cases where the original study supported the alternative but the replication did not, the original study had the larger sample size. In each of the 7 cases where the replication provided strong evidence for the alternative but the original did not, it was the replication that had the larger sample size.

## 4 Discussion

Small sample sizes and underpowered studies are endemic in psychological science. Publication bias is the law of the land. These two weaknesses of our field have conspired to create a literature that is rife with false alarms [27]. From a Bayesian reanalysis of the Reproducibility Project: Psychology, we conclude that one reason many published effects fail to replicate appears to be that the evidence for their existence was unacceptably weak in the first place.

Crucially, our analysis revealed no obvious inconsistencies between the original and replication results. In no case was an hypothesis strongly supported by the data of one team but contradicted by the data of another. In fact, in 75% of cases the replication study found qualitatively similar levels of evidence to the original study, after taking into account the possibility of publication bias. In many cases, one or both teams provided only weak or ambiguous evidence, and whenever it occurred that one team found strong evidence and the other did not, this was easily explained by (sometimes large) differences in sample size. The apparent discrepancy between the original set of results and the outcome of the Reproducibility Project can be adequately explained by the combination of deleterious publication practices and weak standards of evidence, without recourse to hypothetical hidden moderators.

The Reproducibility Project: Psychology is a monumental effort whose preliminary results are already transforming the field. We conclude with the simple recommendation that, whenever possible, empirical investigations in psychology should increase their planned replication sample sizes beyond what is implied by power analyses based on effect sizes in the literature. Our analysis in that sense echoes that of Fraley and Vazire [28].

Decades of reliance on orthodox statistical inference—which is known to overstate the evidence against a null hypothesis [29–32]—have obfuscated the widespread problem of small samples in psychological studies in general and in replication studies specifically. While 92% of the original studies reached the statistical significance threshold (*p* <.05), only 43% met our criteria for strong evidence, with that number shrinking further to 26% when we took publication bias into account. Furthermore, publication bias inflates published effect sizes. If this inflationary bias is ignored in prospective power calculations then replication attempts will systematically tend to be underpowered, and subsequently will systematically obtain only weak or ambiguous evidence. This appears to have been the case in the Reproducibility Project: Psychology.

A major selling point of Bayesian statistical methods is that sample sizes need not be determined in advance [33], which allows analysts to monitor the incoming data and stop data collection when the results are deemed adequately informative; see Wagenmakers et al. [34] for more detail and see Matzke et al. [35] for an implementation of this kind of sampling plan, and also see Schönbrodt et al. [36] for a detailed step-by-step guide and discussion of this design. Subsequently, if the planned sample size is reached and the results remain uninformative, more data can be collected or else researchers can stop and simply acknowledge the ambiguity in their results. Free and easy-to-use software now exists that allows this brand of sequential analysis (e.g., JASP [37]).

This is the first of several retrospective analyses of the Reproducibility Project data. We have focused on a subset of the reproduced studies that are based on univariate tests in order to account for publication bias. Other retrospectives include those that focus on Bayes factors and Bayesian effect size estimates [38].

## Supporting Information

S1 Table Table. Inferential statistics for each of the 72 studies and their replicates.
(PDF)
