# Supplementary material for: A Bayesian Perspective on the Reproducibility Project: Psychology
Source: PLoS One. 2016 Feb 26;11(2):e0149794. doi: 10.1371/journal.pone.0149794 (PMC4769355; doi:10.1371/journal.pone.0149794)
Supplement: S1 Table — Inferential statistics for each of the 72 studies and their replicates. (PDF) [file pone.0149794.s001.pdf]

Supporting Information

S1 Table

Table. Inferential statistics for each of the 72 studies and their replication attempts.

Table 1: Relevant statistics for each of the 72 included studies. Note that Bayes factors are presented on the $\log_{10}$ scale, so positive values favor $\mathcal{H}_A$ and negative values favor $\mathcal{H}_0$; $|\log_{10}(BF)| > 1$ indicate strong evidence favoring the respective hypothesis.

study	original		replicate		$\log_{10}$ Bayes factor		
number	df	t-value	df	t-value	original	mitigated	replicate
1	13	2.6665	28	0.7937	0.6441	0.1108	-0.3291
2	23	3.7027	23	1.1314	1.5810	0.8406	-0.1752
3	24	2.3000	31	1.2272	0.4905	-0.0513	-0.1799
4	190	3.2388	268	0.1000	1.3270	0.5640	-0.8735
5	31	2.8948	47	0.9327	0.9802	0.3126	-0.3767
6	23	3.5500	31	2.4000	1.4556	0.7298	0.5672
7	99	10.1800	14	0.4960	13.9830	13.1400	-0.2826
8	37	4.1267	31	0.6197	2.2564	1.4573	-0.3953
10	28	5.1662	29	6.7283	3.0554	2.2314	4.5391
11	21	4.1593	29	2.8397	1.8813	1.1144	0.9241
15	94	1.9290	241	3.9550	0.0730	-0.1539	2.3326
19	31	3.7683	19	1.9134	1.7949	1.0271	0.2423
20	94	2.2294	106	0.2000	0.3236	-0.2641	-0.7108
24	152	4.8141	48	2.0543	3.7866	2.9503	0.2679
26	94	1.5811	92	1.3964	-0.1753	-0.3290	-0.2842
27	31	2.2738	70	3.4326	0.4696	-0.0854	1.6253
28	31	2.0248	90	0.9849	0.2879	0.0638	-0.4829
29	7	2.8920	14	3.7080	0.5192	0.0899	1.2588
32	36	4.7833	37	3.3347	2.9577	2.1352	1.4245
33	39	3.7700	39	2.0800	1.8938	1.1147	0.3089
36	20	4.5596	20	4.1653	2.1323	1.3475	1.8434
37	11	2.1909	17	1.5395	0.3697	0.1730	0.0476
44	67	3.0800	176	2.0160	1.2134	0.4831	0.0398
48	92	-2.2200	192	-0.7255	0.3186	-0.2666	-0.7393
49	34	2.3833	86	0.2828	0.5528	-0.0301	-0.6593
52	131	2.4062	111	0.9950	0.4373	-0.1962	-0.5215
53	31	2.2672	73	0.6573	0.4646	-0.0891	-0.5524
56	99	4.0768	38	-0.2600	2.5232	1.7072	-0.4970
58	182	2.2891	278	0.6132	0.2790	-0.3413	-0.8382
61	108	-2.3400	220	0.0700	0.4038	-0.2116	-0.8509
63	68	2.3495	145	0.8911	0.4744	-0.1317	-0.6207
65	41	3.0659	131	0.1342	1.1730	0.4637	-0.7584
68	116	2.0372	222	0.0447	0.1246	-0.4201	-0.8525
71	373	4.4000	175	0.9730	2.9768	2.1537	-0.6328
72	257	3.4029	247	0.7000	1.5031	0.7229	-0.8005
81	90	2.6420	137	1.1958	0.7253	0.0539	-0.4730

Table 2: Relevant statistics for each of the 72 included studies (cont'd).

study	original		replicate		$\log_{10}$ Bayes factor		
number	df	t-value	df	t-value	original	mitigated	replicate
87	51	3.0757	47	0.0894	1.2009	0.4805	-0.5511
89	26	0.7200	26	0.1500	-0.3374	-0.3756	-0.4331
93	83	3.0500	68	-1.1240	1.1752	0.4430	-0.3673
94	26	1.8700	59	2.3250	0.1981	0.0087	0.4679
97	73	3.4914	1486	1.4248	1.7004	0.9244	-0.9051
106	34	2.4083	45	1.5340	0.5730	-0.0149	-0.0775
107	84	2.0900	156	1.3180	0.2209	-0.3317	-0.4358
110	278	11.1077	142	1.0909	20.9560	20.1120	-0.5321
111	55	2.6230	116	2.4960	0.7462	0.0937	0.5443
112	9	2.9496	9	3.4059	0.6473	0.1493	0.8169
113	124	10.3600	175	15.6400	15.4490	14.6050	31.4220
114	30	3.8066	30	4.7191	1.8159	1.0472	2.7092
115	31	3.2300	8	-1.4260	1.2825	0.5684	0.0489
116	172	3.9400	139	4.0200	2.3526	1.5385	2.4698
118	111	2.3046	158	0.6156	0.3665	-0.2416	-0.7251
120	29	2.2123	41	1.6533	0.4258	-0.1123	0.0091
122	7	2.7600	16	-9.5900	0.4803	0.0636	3.8735
124	34	2.4269	68	0.2828	0.5880	-0.0035	-0.6110
127	28	4.9800	25	-3.1030	2.8817	2.0618	1.1170
129	26	2.0421	64	0.1414	0.3105	0.0892	-0.6110
133	23	2.3875	37	2.8425	0.5513	-0.0038	0.9505
134	115	2.3030	234	8.8360	0.3596	-0.2489	13.5090
135	562	-0.1100	3511	-6.3100	-0.9042	-0.9044	5.8207
136	28	3.0400	56	-0.7700	1.0900	0.4085	-0.4664
145	76	10.4757	36	5.1730	13.1250	12.2820	3.3895
146	14	3.2000	11	1.9000	0.9709	0.3512	0.2339
148	194	2.6758	259	0.4858	0.6592	-0.0370	-0.8442
149	194	2.6758	314	0.3240	0.6592	-0.0370	-0.8799
150	13	3.7683	18	0.9000	1.2348	0.5677	-0.2222
151	41	2.7946	124	0.0316	0.9115	0.2428	-0.7509
153	7	4.4500	7	0.3200	0.8838	0.3464	-0.2037
154	68	3.9275	14	0.4141	2.2479	1.4437	-0.2958
155	51	2.3286	70	0.2846	0.4848	-0.1069	-0.6167
158	38	2.4920	93	4.3520	0.6405	0.0299	2.9206
161	44	3.6633	44	1.1987	1.8164	1.0407	-0.2521
167	17	3.0545	21	1.2042	0.9613	0.3306	-0.1315
